# A Randomized Controlled Trial Evaluating Integrative Psychotherapeutic Group Treatment Compared to Self-Help Groups in Functional Vertigo/Dizziness

**DOI:** 10.3390/jcm10102215

**Published:** 2021-05-20

**Authors:** Karina Limburg, Katharina Radziej, Heribert Sattel, Peter Henningsen, Marianne Dieterich, Thomas Probst, Rachel Dale, Claas Lahmann

**Affiliations:** 1 Department of Psychosomatic Medicine and Psychotherapy, Klinikum rechts der Isar, Technical University Munich, 81675 Munich, Germany; k.radziej@tum.de (K.R.); h.sattel@tum.de (H.S.); p.henningsen@tum.de (P.H.); 2 German Center for Vertigo and Balance Disorders, Ludwig-Maximilians-University, University Hospital, 81377 Munich, Germany; marianne.dieterich@med.uni-muenchen.de; 3 Department of Neurology, Ludwig-Maximilians-University, University Hospital, 81677 Munich, Germany; 4 Cluster of Systems Neurology-SyNergy, 81377 Munich, Germany; 5 Department for Psychotherapy and Biopsychosocial Health, Danube University Krems, 3500 Krems, Austria; thomas.probst@donau-uni.ac.at (T.P.); rachel.dale@donau-uni.ac.at (R.D.); 6 Department of Psychosomatic Medicine and Psychotherapy, Faculty of Medicine, Medical Center-University of Freiburg, 79104 Freiburg, Germany

**Keywords:** psychotherapy, vertigo, dizziness, randomized controlled trial

## Abstract

We tested the efficacy of an integrative psychotherapeutic group treatment (IPGT) in reducing vertigo/dizziness-related impairment along with depression, anxiety, and somatization by conducting a randomized controlled superiority trial comparing IPGT to self-help groups moderated by a clinical psychologist (SHG). Adult patients with functional vertigo and dizziness symptoms were randomly allocated to either the IPGT or SHG as active control group. Outcomes were assessed at baseline (t0), after treatment lasting 16 weeks (t1), and 12 months after treatment (t2). A total of 81 patients were assigned to IPGT and 78 patients were assigned to SHG. Vertigo-related impairment was reduced in both conditions (IPGT: t0–t1: d = 1.10, t0–t2: d = 1.06; SHG: t0–t1: d = 0.86, t0–t2: d = 1.29), showing the efficiency of both IPGT and SHG. Clinically relevant improvements were also obtained for depression in both groups. Linear mixed model analyses revealed no differences between groups for all outcomes (effect of group for the primary outcome: b = −1.15, SE = 2.13, t = −0.54, *p* = 0.59). Attrition rates were higher in SHG (52.6%) than in IPGT (28.4%). Both conditions improved primary and secondary outcomes while IPGT was better accepted by patients than SHG. Trial registration: ClinicalTrials.gov, Identifier: NCT02320851.

## 1. Introduction

With a lifetime prevalence of up to 30% and a one-year prevalence of 23–25%, vertigo and dizziness are frequent symptoms [1,2,3,4]. Although various organic disorders may be underlying, a substantial number of patients presented without any detectable structural dysfunction and therefore suffer from functional vertigo and dizziness. The term “functional vertigo and dizziness” has been defined and supported by Dieterich and Staab (2017) [5] as the new nomenclature to refer to one and the same construct that had previously been given very different terms such as somatoform dizziness, phobic postural vertigo, or persistent postural–perceptual dizziness.

Functional vertigo and dizziness symptoms frequently occur comorbidly with anxiety, depressive, or somatoform and somatic symptom disorders [6,7,8]. Longitudinal studies show that dizziness can be a risk factor for developing depression [9] and that depression, anxiety, and somatoform disorders can worsen the distress experienced by vertigo and dizziness symptoms [10].

Although functional vertigo and dizziness symptoms are highly prevalent and severely impairing conditions, currently existing treatment options are unsatisfactory. Results from a systematic review including three randomized controlled trials indicated that cognitive behavioural therapy (CBT) together with relaxation techniques or vestibular rehabilitation may moderately reduce dizziness-related burden but not anxiety or depression [11]. 

Similar results occurred in another RCT that compared a three-session CBT intervention based on the CBT model of panic disorder to a waitlist control group. Whilst there were significant and substantial reductions in dizziness-related symptoms that remained stable up to six-month follow-up, depression and anxiety did not change [12,13]. Another controlled study compared a psychoeducative group program including relaxation and balancing exercises to a wait-list control group. The treatment group reported higher decreases in dysfunctional illness representations and better increases in patient empowerment than the control group, with results remaining stable over the 3- and 12-month follow-up periods. However, changes in anxiety, depression, and somatisation were very small and not significant [14]. More recently, Schmid and colleagues [15] found an eight-session cognitive–behavioral group therapy effective in improving vertigo-related impairment in dizziness patients without quantified balance deficits and similarly, Toshishige et al. [16] found that a five-session group CBT therapy significantly reduced dizziness in chronic subjective dizziness patients. Acceptance and commitment therapy and vestibular rehabilitation have also shown some initial promise for persistent and chronic dizziness [17,18]. 

Hence, although there is some evidence for the efficacy of current therapeutic programs, there is a need for improvement. Existing literature on the topic seems scarce, especially with regard to the effectiveness of different forms of therapy (see [19,20]). Dizziness is usually the symptom, but not the actual disorder; thus, the disorder needs to be addressed, which becomes evident with a gaze toward comorbidity. Due to frequently comorbid psychological disorders, it is insufficient to treat merely the vertigo and dizziness symptoms, but it is necessary to also address the accompanying symptoms of depression, anxiety, and somatization. This is especially important due to their role as maintaining factors that worsen the burden caused by the vertigo and dizziness symptoms themselves [10]. 

Therefore, the aim of the current study was to test the efficacy of a newly developed integrative psychotherapeutic group intervention (IPGT) that is tailored to both the functional vertigo and dizziness symptoms as well as the frequent comorbidities of anxiety, depression, and somatization. This new intervention was compared to self-help groups (SHG) that served as an active control condition controlling for unspecific change mechanisms such as therapeutic relationship, group cohesion, and receiving professional attention. 

Firstly, it was hypothesized that IPGT results in significant and clinically relevant changes in all primary and secondary outcomes in the short and long term (after 16 weeks of treatment and at 12-month follow-up) and, secondly, that it is superior in improving all outcomes compared to the active control group. In addition, we aimed to conduct exploratory investigations of further predictors of change next to group: namely, symptom duration, a depressive or anxiety disorder at baseline, and previous psychotherapy. The rationale, aims, hypotheses, and methods of the current study have been described previously in the respective study protocol [21]. The IPGT intervention has been tested in a pilot trial that found small to large effects of IPGT on primary and secondary outcome measures. Acceptance and feasibility of the treatment concept was supported in this trial [22].

To the best of our knowledge, the study is the first trial investigating the efficacy of a manualized group treatment program based on principles of integrative psychotherapy and tailored to functional vertigo and dizziness symptoms and common comorbid disorders compared to moderated self-help groups.

## 2. Materials and Methods

### 2.1. Study Design

**Figure 1:**
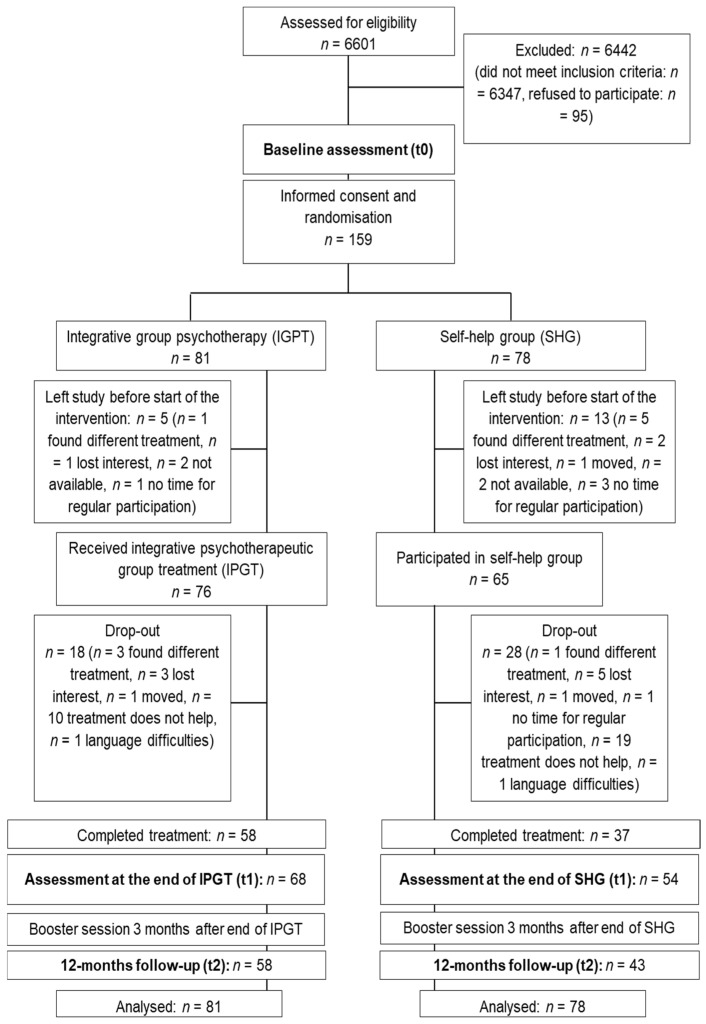
Study design and patient flow.

As described in the study protocol [21], the trial was designed as a two-armed single-center randomized controlled open clinical superiority trial with a parallel active control intervention and balanced randomization (1:1). There were three assessment points, at baseline (t0), at the end of treatment (t1), and 12 months after the end of treatment (t2). The study design is presented in Figure 1. The study was registered under ClinicalTrials.gov with the trial identifier NCT02320851.

### 2.2. Participants

Patients were recruited via routine care appointments at the German Centre for Vertigo and Balance Disorders at the University Hospital Munich between April 2015 and February 2018. The center is a specialized tertiary care unit where patients are referred to by practitioners specialized in neurology, ophthalmology, or ENT. Hence, patients presenting at the center would have been previously seen by doctors from at least two disciplines (general practitioner plus neurologist, ophthalmologist, and/or ENT specialist). All patients underwent structured history assessment and a systematic and standardized physical examination including neurological, neuro-otological, and neuro-ophthalmological examinations. Inclusion criteria were age of 18 years or older (with an average age at entry of M = 53.7 for the experimental and M = 53.5 for the control group) and a diagnosis of functional vertigo or dizziness. This diagnosis is made based on the criteria presented by Dieterich and Staab (2017) [5]. Those criteria include (a) one or more symptom of dizziness, unsteadiness, or nonspinning vertigo that are present on most days for 3 months or more and (b) symptoms being present without specific provocation but exacerbated by upright posture, active or passive motion without regard to direction or position, and/or exposure to moving visual stimuli or complex visual patterns. Further, (c) functional vertigo and dizziness usually begins shortly after an event, causing acute vestibular symptoms or problems with balance. Lastly, (d) symptoms cause significant distress or functional impairment and are (e) not better attributed to another disease or disorder. In accordance with criterion (d), patients had to report significant impairment caused by their complaints as indicated by a sum score of 45 or higher on the Vertigo Handicap Questionnaire. Exclusion criteria were insufficient German language ability, cognitive disorder indicated by a score below 26 points on the Montreal Cognitive Assessment, major impairment of social functioning caused by suicidality, psychosis, substance abuse, and/or other severe mental disorders, and current psychotherapy. Further, patients with permanent residency more than 50 km away from the trial site or other reasons that would make treatment adherence and completion of the study unlikely were excluded. Additional patient characteristic information is presented within Table 1.

### 2.3. Interventions

Patients were randomly assigned to the study (IPGT; *n* = 81), or the active control intervention (SHG, *n* = 78). Patients in both arms received the same dose (16 weekly sessions of 90 minutes) and mode (group setting with 6–10:2 patients to therapist ratio) of intervention and were asked to keep their medication unchanged during the trial period. Patients in the control group, instead of receiving IPGT, received a series of moderated SHGs; thus, the control group was an active one, which has to be considered for interpretation of the results.

An intervention protocol was developed that combined proven therapeutic strategies from previous studies, including psychodynamic–interpersonal therapy for somatoform disorder (PISO), an individual and manualized short-term intervention that has been shown to be effective [23]. Patients in the intervention group received IPGT combining cognitive–behavioral and psychodynamic elements together with psychoeducation and balance control training with physical exercises. This training was conceptualized to offer gradual exposure to movements that are usually avoided and is based on findings of increased visual dependence for spatial orientation and postural control in patients with functional vertigo and dizziness [24,25,26,27,28]. The treatment design, with its three treatment phases, is presented in Table 2, with further details provided in the study protocol [21] and pilot study [22].

Patients in the active control group took part in SHGs that were moderated by psychologists in clinical training. Moderators were instructed not to offer any therapeutic interventions and instead only guide through the sessions. Hence, SHGs were offered as an active control condition in order to ensure study participation and for ethical reasons. Further details are provided elsewhere [21]. 

### 2.4. Assessments and Outcomes

**Clinical assessment.** Next to the medical assessment described above, all participants underwent a clinical interview using the Clinical Interview for DSM-IV [29] because the German version of the respective interview for DSM-5 was not available at the time. 

**Sociodemographic assessment.** We assessed participants’ gender, age, education, and the duration of complaints. Furthermore, we asked whether participants had previously been in any form of psychotherapeutic treatment.

**Primary outcomes.** To assess our primary outcome, vertigo-related impairment, or impairment, we used the Vertigo Handicap Questionnaire (VHQ; [30,31]). The VHQ assesses physical and psychosocial impairments associated with the symptoms with 25 items scored on a 5-point Likert scale and allows for a sum score (range: 0–100) and two subscale scores: vertigo-related anxiety (VHQ-ANX; range: 0–4) and restriction of activity (VHQ-ACT; range: 0–4). Higher scores indicate higher impairment. 

**Secondary outcomes.** The Vertigo Symptom Scale (VSS; [32]), with the two subscales vertigo and associated symptoms (VSS-VER) and somatic anxiety and autonomic arousal (VSS-AA), was used to assess the frequency of dizziness-related symptoms using 34 Likert-style items. 

To assess somatization, we applied the Patient Health Questionnaire (PHQ-15; [33]), which is a 15-item instrument that allows sum scores ranging from 0 to 30. 

The Beck Anxiety Inventory [34] was used to assess anxiety severity during the last seven days with 21 items that are answered on four-point Likert scales from 0 to 3. 

We used the revised edition of the Beck Depression Inventory [35] to assess depression severity during the last two weeks. It consists of 21 items with four-point Likert scales ranging from 0 to 3. 

The Short-Form Health Survey (SF-12; [36,37]) was used to measure both physical and psychological health-related quality of life (HRQoL). 

### 2.5. Sample Size

The details of the sample size calculation are described in the study protocol [21]. An improvement of 10 points over time per patient was regarded as being clinically relevant. Based on this, the detectable difference per group was estimated as a standardized effect size of 0.5. A power calculation with a statistical power of 90% and a type I error probability of 0.05 for a two-sided t-test resulted in a sample size of 86 patients per study arm. We were able to include a total of 159 patients (IPGT: *n* = 81, SHG: *n* = 78). Since we observed effect sizes that were higher than expected (IPGT: Cohen’s d = 1.05, SHG: Cohen’s d = 1.29), our analyses were nevertheless sufficiently powered (statistical power > 90%). 

### 2.6. Randomization and Blinding

Randomization, using randomly selected balanced blocks with block sizes of 2, 4, or 6 patients, was balanced (1:1) and conducted directly after assessing eligibility and obtaining informed consent to participate. The random allocation sequence was generated by a senior researcher who was not in contact with patients and did not conduct the treatment sessions (HS). After assessing eligibility, conducting the clinical interview and obtaining informed consent, one of two researchers (KR or KL) opened an envelope that contained the randomized treatment allocation. In this way, they were blinded to the patient’s group allocation while conducting the initial assessment. Blinding of therapists (KR and KL) and moderators of the self-help groups was not possible once a patient was allocated to an intervention group. 

### 2.7. Ethical Issues

The trial has been reviewed and approved by the Ethics Committee of the Medical Department of the LMU Munich (ref: 319-14). Safety issues were handled in line with the procedures described in the study protocol [21]. The trial was conducted according to the Guideline for Good Clinical Practice and the declaration of Helsinki. 

### 2.8. Statistical Analyses

Two sets of analyses were conducted to compare (a) the treatment groups and (b) those who had completed treatment vs. patients that dropped out during the course of the study, regarding differences in sociodemographic and clinical characteristics as well as their baseline scores on the primary and secondary outcome measures. In addition, effect sizes (Cohen’s *d*) were calculated. Effect sizes were interpreted in line with Cohen’s rules of thumb, with 0.2 < *d* < 0.5 indicating a small, 0.5 ≤ *d* < 0.8 indicating a medium, and *d* ≥ 0.8 indicating a large effect [38]. Furthermore, effect sizes were interpreted according to the minimal clinically important difference (MCID), indicating whether a change in symptom intensity can be perceived for a patient and therefore be considered clinically meaningful. An effect size between 0.3 and 0.5 can be considered clinically meaningful [39]. 

After this, linear mixed models were conducted to model the primary and secondary outcomes over the time periods from baseline to the end of treatment (t0–t1) and from baseline to 12-months follow-up (t0–t2). This multilevel modeling approach is recommended especially for analyzing longitudinal data with a nested structure and potential missings due drop-outs in repeated measurements (assessments nested within patients) [40]. Predictor variables were time, group, the interaction between time and group, drop-out during treatment, age, gender, education, symptom duration, depressive and anxiety disorders, and previous psychotherapy. Time was dummy-coded in order to represent both the time period from baseline to the end of treatment and from baseline to 12-months follow-up with one separate variable per time period. To analyze the predictive value of these variables, we compared five different models by performing likelihood-ratio tests: The baseline model contained two fixed main effects for the time periods and a random intercept for participants. This baseline model was compared to four models that add (1) a by-participant random slope for each time period; (2) a fixed main effect for treatment and a by-participant random slope for each time period; (3) a fixed main effect for treatment, a fixed interaction effect between treatment and each time period, and a by-participant random slope for each time period, and (4) a fixed main effect for treatment, a fixed interaction effect between treatment and each time period, and a by-participant random slope for each time period, as well as the covariates age, gender, education, duration of symptoms, baseline diagnoses of depression and anxiety, previous psychotherapeutic treatment, and drop-out during the study. The current study presents the full model with all covariates included, since it analyses the full range of hypothesised covariates. 

Data were analyzed using IBM SPSS 24.0 [41] and the lme4 package for *R* [42]. As per the default setting in lme4, the covariance structure in the linear mixed model analyses was set as unstructured. 

## 3. Results

A total of 159 patients gave their informed written consent and were randomly allocated to IPGT (*n* = 81) or SHG (*n* = 78); 18 left the study before the start of the interventions (Figure 1). Initial analyses showed that both groups, IPGT and SHG, differed significantly regarding educational level, previous psychotherapy, and drop-out rates during the course of the intervention (Table 2 and Table 3). With attrition rates of 28.4% for IPGT and 52.6% for SHG, the difference in drop-out rates between the groups was high and statistically significant (*Χ*^2^ = 9.65, *p* = 0.002). 

### 3.1. Changes in Primary and Secondary Outcomes

Table 3 shows the mean values of the primary (vertigo-related impairment, VHQ) and secondary outcomes at the three measurement time points. Effect sizes for the differences between groups were small. Regarding the within-group difference between measurement time points, effect sizes were large for the primary outcome and small to medium for the remaining outcomes in both groups. Considering MCID, effects were clinically meaningful for the VHQ sum score and its subscales in both groups. For most secondary outcomes, we observed clinically meaningful differences for the time period from baseline to 12-month follow-up but not for baseline to treatment completion. For somatization, physical HRQoL, and anxiety, the effect was clinically meaningful only in the control group.

### 3.2. Linear Mixed Model Analyses

Table 4 presents the results of the linear mixed model analyses. For the time period from baseline to follow-up (T0–T2), effects of time were significant for all outcomes except for mental HRQoL. However, this improvement over time did not differ between groups, as displayed by the non-significant interaction effects between time period (T0–T2) and group for all outcomes except for physical HRQoL. Here, the negative significant interaction effect indicated that improvements were higher in the control group compared to the treatment group. This may partly be explained by the non-significant but large difference between the groups on physical HRQoL. 

For the time period from baseline to after treatment (T0–T1), effects of time were significant for the primary outcome, the VHQ sum score as well as the subscales, and somatization (PHQ-15), but not for the remaining outcomes. Interactions between this time period (T0–T1) and group were non-significant throughout. Hence, our hypothesis that patients in IPGT would report significantly higher improvements in primary and secondary outcome measures compared to patients in SHG cannot be confirmed. In terms of other predictors that were investigated, a depressive disorder was significantly associated with higher vertigo-related handicap, mental HRQoL, depression, somatization and anxiety. Previous psychotherapeutic treatment was significantly associated with higher autonomic arousal, lower mental HRQoL, and higher levels of depression and anxiety. A longer symptom duration was positively associated with vertigo severity.

## 4. Discussion

Our aim was to investigate the efficacy of an integrative psychotherapeutic group intervention for patients with functional vertigo and dizziness symptoms by testing whether it is superior to the active control condition, a moderated self-help group. Our first hypothesis that IPGT would result in clinically relevant improvements over the 12-month follow-up period can be confirmed for the primary outcome, vertigo-related handicap, restriction of activity, and vertigo-related anxiety and secondary outcomes vertigo severity, autonomic arousal, mental HRQoL, and depression. Clinically relevant improvements were not attained for secondary outcomes somatisation, physical HRQoL, and anxiety. Hence, patients did not only improve in outcome measures directly related to their complaints, but they also finished treatment with lower depression scores and increased mental HRQoL in the long-term. A long-term improvement in depression was not achieved in previous studies [11,12,13,14]. However, although we had also aimed to improve anxiety and somatization scores, differences were not clinically meaningful in IPGT.

We cannot confirm our second hypothesis stating that IPGT would be superior to SHG, which was used as an active control condition, in improving primary and secondary outcomes. Instead, we observed similar and for physical HRQoL even higher effects for SHG than for IPGT. This finding is surprising in the light of diverging results of previous treatment studies with active control groups that reported significantly better effects for the treatment group [11]. To the best of our knowledge, the current study is the first applying an active control group in the same dose and setting as the intervention group, thus using the same number of meetings and the overall same intensity, in order to make for a perfect comparability. The self-help groups were moderated by psychologists during their clinical training. Although they did not offer specific interventions, the presence of an expert certainly had effects—such as the fact that patients receive professional time, attention, and cognitive stimulation (see [43]). Furthermore, considering that the same two moderators were present over the whole treatment period, thereby providing stability and establishing a friendly atmosphere, a therapeutic alliance was built up, although no specific therapeutic interventions were delivered. Therefore, our results may support findings suggesting that therapeutic alliance as an unspecific treatment factor predicts symptom improvement [44,45] and thereby adds to the debate on the role of common vs. specific factors in general [46]. 

Our findings raise the question of whether the improvement in our sample represents merely a spontaneous remission that would have occurred even without any form of treatment. On the one hand, this is unlikely, considering previous findings of our group suggesting that vertigo and dizziness symptoms often turn into chronic conditions if treated as usual [7,47]. By contrast, other findings point out that patients often benefit from a thorough medical examination along with a brief psychoeducation about their symptoms. In a follow-up study involving 78 patients, 72% were free of symptoms or exhibited a clear improvement 0.5–5.5 years after the initial diagnosis of functional vertigo and dizziness after a thorough diagnostic evaluation to demonstrate to patients that their symptoms are of functional origin and they do not have an active structural disorder, education about the nature of the disorder, and desensitization by self-exposure to triggers and regular physical therapy [48]. Another longitudinal follow-up study (5–16 years) on 106 patients treated with the same strategy showed that dizziness symptoms improved at a rate of 75%; the symptoms had fully resolved in 27% [49]. Hence, one may conclude that even patients treated as usual would show an improvement. In this regard, comparing IPGT to a waitlist or treatment as usual control condition would be an important step for future research. 

Our findings make it seem worthwhile to further investigate beneficial aspects of self-help groups. Evidence suggests that self-help interventions promoting health literacy by instructing patients with medically unexplained symptoms to get informed about their condition and to apply self-management techniques are effective in reducing symptom-related impairment [50]. However, most of the research on self-help techniques excludes self-help groups that allow sharing experiences with others. This is surprising, since a group setting offers group cohesion as an important change factor [51]. So far, few studies have systematically investigated self-help groups. Payne and colleagues [52] compared individualized cognitive treatment to a self-help support group and a waitlist control group for patients with irritable bowel syndrome. While cognitive therapy was superior overall, patients in the self-help group had higher improvements compared to the waitlist control group. Later on, the same authors and co-workers compared group-based cognitive therapy, psychoeducational support groups as an active control, or intensive symptom and daily stress monitoring. Group cognitive therapy and psychoeducational support groups were statistically superior and continued not to differ on any measure at follow-up [53].

Despite all potential benefits of self-help groups, it is important to emphasize that drop-out rates were significantly higher in SHG compared to IPGT. Thus, patient acceptance was higher in the treatment group. Therefore, while both conditions provided clinical benefits, IPGT still seems to be the favorable approach, as drop-out rates and client satisfaction are relevant indicators within the practical work. The good acceptance of IPGT may have occurred because we did not offer purely psychotherapeutic treatment but incorporated psychophysiological elements. Similar conclusions were drawn by Katsamanis and colleagues [54], who treated patients with various medically unexplained symptoms with a psychophysiological intervention that incorporated physiological treatment elements such as biofeedback. Patients in the intervention group had significantly greater improvements than those receiving standard medical care. Authors suggested that observably “medical” interventions would be more attractive to their patient group who are often convinced that their symptoms are caused by an organic dysfunction. Furthermore, vestibular rehabilitation was found to be effective in patients with functional vertigo in a retrospective review [55]. A recent trial combined vestibular rehabilitation and cognitive behavior therapy and found it to be feasible for people with persistent dizziness, and acceptance amongst participants and therapists was high [43]. The same may apply to our patients. Our patients underwent a standardized and clearly defined treatment with every session starting with physical exercises to stimulate the vestibular system. Furthermore, the first sessions contained several psychoeducative elements explaining the interaction between biological, social, and psychological aspects. Hence, although the treatment focused on psychotherapeutic interventions, the physiological aspects were well considered, thereby catering to patients’ desire to consider the structural aspects of their complaints and heightening acceptance. 

Next to evaluating clinical improvements, our findings should also be considered from a cost–benefit perspective. Meta-analytic evidence suggests that group interventions for patients with medically unexplained symptoms might be more cost-effective than those delivered in an individual setting [56]. This seems valid for the current trial as well, since within-group effect sizes are comparable to trials evaluating individual interventions [12,23]. If SHG could again prove its ability to improve relevant clinical outcomes, it would be even more cost-effective than IPGT in a group setting, since it does not need to be delivered by trained clinical psychologists but by trained health staff such as nurses or final-year students of clinical psychology instead. 

There were limitations. One was that that our sample consists of patients who have already had consultations in primary and secondary care since we recruited at a specialized tertiary care center. Therefore, the duration of patients’ complaints is likely much longer than what one would see in primary or secondary care. Limitations further include that we were unable to recruit the full sample of 216 patients as originally planned. This was mainly due to the fact that patients were often living more than 50 km away from the trial site, making it hard for them to regularly attend group sessions. Further, drop-out rates during treatment were high with 28.4% for IPGT and 52.6% for SHG. This was accounted for in our analysis by using multilevel modeling, which is an approach recommended for longitudinal data with a nested structure and potential drop-out rates during follow-up assessments. In addition, the study did not show clear evidence for the increased efficiency of IPGT over SHG, and a control group would be needed to determine the extent of improvement for each intervention; however, it could be shown that both approaches can be used in a similarly efficient manner. 

## 5. Conclusions

In summary, we observed substantial and long-lasting improvements in the primary and nearly all secondary outcome measures assessing vertigo-related impairment, vertigo, symptom severity, mental HRQoL, and depression. Unexpectedly, we did not see superior effects of IPT compared to SHG. Our results serve as evidence for potentially beneficial aspects of SHGs for patients that often do not get in touch with others who suffer from similar complaints. Better support for patients in establishing networks may help to improve patient outcome. In favor of IPGT, patient acceptance was considerably higher compared to SHG. This may indicate that IPGT is more efficacious than SHG, assuming that patients who terminated the trial prematurely did not experience a significant improvement. 

It is also the first study finding significant effects in reducing not only the vertigo-related symptoms themselves but also depression. We assessed long-term effects and found stable improvements. The active control condition allowed us to control for non-specific change mechanisms such as the therapeutic relationship, group cohesion, and professional attention paid to the participants.

**Summary box**

- We investigated the efficacy of an integrative psychotherapeutic group intervention (IPGT) for patients with functional vertigo and dizziness symptoms in comparison to an active control condition, a moderated self-help group (SHG).
- Results indicate substantial and long-lasting improvements in the primary and nearly all secondary outcome measures assessing vertigo-related impairment, vertigo, symptom severity, mental HRQoL, and depression in both groups.
- Unexpectedly, we did not see superior effects of IPGT compared to SHG. Hence, our results point at the potentially beneficial aspects of SHGs for our patient group. Therefore, assisting patients in establishing networks with others suffering from the same or similar condition may help to improve patient outcome.
- It should be noted that while drop-out rates were generally high, patient acceptance was considerably higher in IPGT compared to SHG.

## Figures and Tables

**Table 1:** Patient characteristics at baseline.

	IPGT (*n* = 81)	SHG (*n* = 78)	*t* or *Χ*^2^	*p*
Age at entry, *M* (*SD*)	53.7 (15.4)	53.5 (15.1)	*t* = −0.11	0.917
Female, *n* (*%*)	49 (60.5)	49 (62.8)	*Χ*^2^ = 0.09	0.871
Education, *n* (*%*)			*Χ*^2^ = 9.89	0.042
9th grade or less	20 (24.7)	14 (17.9)		
10th grade	25 (30.9)	19 (24.4)		
High school graduate	14 (17.3)	17 (21.8)		
University graduate	16 (19.7)	17 (21.8)		
Missing information	6 (7.4)	11 (14.1)		
Married, *n* (*%*)	33 (40.7)	42 (53.8)	*Χ*^2^ = 6.08	0.108
Employment status, *n* (*%*)			*Χ*^2^ = 4.21	0.648
Employed	42 (51.9)	40 (51.3)		
Unemployed	3 (3.7)	3 (3.8)		
Retired	24 (29.6)	22 (28.3)		
Other	12 (14.8)	13 (16.6)		
Psychiatric diagnosis, *n* (*%*)				
Depressive disorder	19 (23.5)	17 (21.8)	*Χ*^2^ = 0.06	0.802
Anxiety disorder	38 (46.9)	34 (43.6)	*Χ*^2^ = 0.18	0.674
Other	9 (11.1)	9 (11.5)	*Χ*^2^ = 0.01	0.932
Symptom duration, *n* (*%*)			*Χ*^2^ = 3.07	0.547
<1–3 months	12 (14.8)	6 (7.7)		
3 months–2 years	36 (44.4)	39 (50.0)		
2–10 years	28 (34.6)	28 (35.9)		
>10 years	5 (6.2)	5 (6.4)		
Previous psychotherapy, *n* (*%*)	33 (40.7)	16 (20.5)	*Χ*^2^ = 5.75	0.017
Drop-out, *n* (%)	23 (28.4)	41 (52.6)	*Χ*^2^ = 9.65	0.002

**Table 2:** Overview of IPGT treatment phases and contents.

Phase	Sessions	Details
1	1–4	Building up of therapeutic relationship through symptom-oriented exploration and psychoeducation regarding psychophysiology of SVD, dysfunctional cognitions, and avoidance behavior Introducing elements of balance control training Elaborating individualized therapy goals for each patient
2	5–13	Broadening the patients’ explanatory model and symptom management Clarification of interpersonal symptom contexts and accompanying affects → differentiation of emotions as well as bodily feelings Improvement of self-regulation Symptom-oriented modules with focus on dysfunctional cognitive and interactional patterns Tailored modules focusing on anxiety/phobic, somatoform, and depressive symptoms
3	14–16	Transfer to daily life Termination

Note: Reprinted from “Tailored care for somatoform vertigo/dizziness: study protocol for a randomized controlled trial evaluating integrative group psychotherapy”, by Lahmann, C., Henningsen, P., Dieterich, M., Radziej, K., Schmid, G. [21].

**Table 3:** Outcome measure scores at baseline, end of treatment, and 12-month follow-up, along with sensitivity analyses comparing the groups at baseline.

	IPGT *M* (*SD*)	SHG *M* (*SD*)	Group Comparison at Baseline		Effect Sizes (Cohen’s *d*)				
			*t*	*p*	IPGT vs. SHG	IPGT: T0–T1	IPGT: T0–T2	SHG: T0–T1	SHG: T0–T2
Vertigo-related handicap (VHQ sum score)						1.10	1.06	0.86	1.29
Baseline	57.34 (10.56)	59.13 (12.04)	0.99	0.32	0.16				
End of treatment	41.65 (17.16)	45.54 (18.76)			0.23				
12-months follow-up	39.95 (20.55)	37.28 (20.77)			0.13				
Restriction of activity (VHQ-ACT)						0.95	0.97	0.83	1.24
Baseline	2.21 (0.49)	2.32 (0.47)	1.48	0.14	0.24				
End of treatment	1.60 (0.75)	1.78 (0.80)			0.23				
12-months follow-up	1.53 (0.87)	1.44 (0.89)			0.11				
Vertigo-related anxiety (VHQ-ANX)						0.81	0.77	0.45	0.86
Baseline	2.61 (0.68)	2.66 (0.78)	0.45	0.66	0.07				
End of treatment	2.02 (0.78)	2.28 (0.93)			0.31				
12-months follow-up	1.93 (1.05)	1.87 (1.04)			0.06				
Vertigo severity (VSS-VER)						0.20	0.72	0.05	0.44
Baseline	1.30 (0.89)	1.23 (0.69)	−0.47	0.64	0.09				
End of treatment	1.13 (0.84)	1.19 (0.79)			0.07				
12-months follow-up	0.75 (0.63)	0.89 (0.82)			0.19				
Autonomic Arousal (VSS-AA)						0.15	0.35	0.15	0.38
Baseline	1.52 (0.85)	1.47 (0.77)	−0.34	0.73	0.06				
End of treatment	1.39 (0.81)	1.35 (0.87)			0.05				
12-months follow-up	1.23 (0.85)	1.16 (0.85)			0.08				
Somatization (PHQ-15)						0.09	0.07	0.28	0.37
Baseline	12.27 (5.31)	12.38 (4.76)	−0.13	0.89	0.02				
End of treatment	11.0 (5.13)	10.76 (6.04)			0.04				
12-months follow-up	10.84 (5.67)	9.95 (6.03)			0.15				
Physical HRQoL (SF-12)						0.20	0.01	0.20	0.58
Baseline	40.08 (8.31)	36.74 (9.58)	−1.83	0.07	0.37				
End of treatment	41.85 (9.55)	38.75 (10.14)			0.31				
12-months follow-up	40.22 (10.35)	42.83 (11.42)			0.24				
Mental HRQoL (SF-12)						0.34	0.43	0.11	0.34
Baseline	40.16 (10.54)	41.63 (13.46)	0.60	0.55	0.12				
End of treatment	43.85 (10.96)	43.05 (12.64)			0.07				
12-months follow-up	44.67 (10.49)	45.94 (11.57)			0.12				
Depression (BDI-II)						0.26	0.39	0.33	0.42
Baseline	15.51 (8.75)	16.97 (11.15)	0.88	0.38	0.15				
End of treatment	13.25 (8.92)	13.56 (9.80)			0.03				
12-months follow-up	12.36 (7.20)	12.26 (11.34)			0.01				
Anxiety (BAI)						0.21	0.26	0.19	0.41
Baseline	19.27 (12.10)	18.69 (11.79)	−0.29	0.77	0.05				
End of treatment	16.78 (11.53)	16.40 (12.08)			0.03				
12-months follow-up	16.14 (12.07)	13.74 (12.24)			0.20				

Note: VHQ = Vertigo Handicap Questionnaire, VSS = Vertigo Symptom Scale, PHQ-15 = Patient Health Questionnaire-15, SF-12 = Short Form Health Survey-12, BDI-II = Beck Depression Inventory-II, BAI = Beck Anxiety Inventory, HRQoL = Health-related quality of life.

**Table 4:** Results of the linear mixed model analyses.

Outcome	Predictors	*b*	*SE*	*t*	Lower	Upper	*p*
Vertigo−related handicap (VHQ sum score)	**Time: T0–T1**	**−11.99**	**2.08**	**−5.77**	**−16.06**	**−7.92**	**<0.001**
	**Time: T0–T2**	**−21.55**	**2.59**	**−8.32**	**−26.63**	**−16.48**	**<0.001**
	Group	−1.15	2.13	−0.54	−5.32	3.03	0.59
	Time (T0–T1) x group	−4.00	2.81	−1.43	−9.51	1.50	0.16
	Time (T0–T2) x group	4.15	3.46	1.20	−2.64	10.94	0.23
	Symptom duration	1.17	1.26	0.93	−1.29	3.63	0.35
	**Depressive disorder**	**5.74**	**2.30**	**2.50**	**1.24**	**10.25**	**0.01**
	Anxiety disorder	−0.05	2.16	−0.02	−4.28	4.18	0.98
	Previous psychotherapy	2.20	2.25	0.98	−2.21	6.61	0.33
Restriction of activity (VHQ−ACT)	**Time: T0–T1**	**−0.49**	**0.09**	**−5.61**	**−0.67**	**−0.32**	**<0.001**
	**Time: T0–T2**	**−0.86**	**0.11**	**−8.16**	**−1.07**	**−0.65**	**<0.001**
	Group	−0.08	0.09	−0.89	−0.26	0.10	0.38
	Time (T0–T1) x group	−0.14	0.12	−1.17	−0.37	0.09	0.24
	Time (T0–T2) x group	0.19	0.14	1.36	−.08	0.47	0.18
	Symptom duration	0.04	0.05	0.73	−0.07	0.15	0.47
	Depressive disorder	0.17	0.10	1.71	−0.02	0.36	0.09
	Anxiety disorder	−0.09	0.09	−0.94	−0.27	0.09	0.35
	Previous psychotherapy	0.07	0.10	0.67	−0.12	0.25	0.50
Vertigo−related anxiety (VHQ−ANX)	**Time: T0–T1**	**−0.33**	**0.11**	**−3.03**	**−0.54**	**−0.11**	**0.003**
	**Time: T0–T2**	**−0.81**	**0.14**	**−5.93**	**−1.08**	**−0.54**	**<0.001**
	Group	−0.05	0.13	−0.36	−0.30	0.21	0.72
	Time (T0–T1) x group	−0.26	0.14	−1.83	−0.55	0.02	0.07
	Time (T0–T2) x group	0.11	0.18	0.63	−0.24	0.47	0.53
	Symptom duration	0.08	0.07	1.12	−0.06	0.22	0.26
	Depressive disorder	0.24	0.13	1.80	−0.02	0.50	0.07
	Anxiety disorder	0.21	0.12	1.67	−0.04	0.45	0.10
	Previous psychotherapy	0.17	0.13	1.28	−0.09	0.42	0.20
Vertigo severity (VSS−VER)	Time: T0–T1	0.01	0.10	0.13	−0.18	0.21	0.89
	**Time: T0–T2**	**−0.31**	**0.12**	**−2.48**	**−0.55**	**−0.06**	**0.01**
	Group	0.07	0.15	0.47	−0.22	0.36	0.64
	Time (T0–T1) x group	−0.13	0.13	−0.97	−0.39	0.13	0.33
	Time (T0–T2) x group	−0.22	0.17	−1.31	−0.55	0.11	0.19
	**Symptom duration**	**0.18**	**0.08**	**2.40**	**0.04**	**0.34**	**0.02**
	Depressive disorder	0.10	0.14	0.70	−0.18	0.37	0.49
	Anxiety disorder	−0.14	0.13	−1.04	−0.39	0.11	0.30
	Previous psychotherapy	0.18	0.14	1.30	−0.09	0.44	0.20
Autonomic Arousal (VSS−AA)	Time: T0–T1	−0.07	0.08	−0.87	−0.23	0.09	0.38
	**Time: T0–T2**	**−0.29**	**0.09**	**−3.05**	**−0.47**	**−0.10**	**0.003**
	Group	0.02	0.13	0.13	−0.25	0.28	0.90
	Time (T0–T1) x group	−0.03	0.11	−0.25	−0.24	0.19	0.81
	Time (T0–T2) x group	0.03	0.13	0.26	−0.21	0.28	0.80
	Symptom duration	0.05	0.08	0.60	−0.11	0.20	0.55
	Depressive disorder	0.18	0.15	1.22	−0.11	0.47	0.23
	Anxiety disorder	0.09	0.14	0.65	−0.18	0.36	0.52
	**Previous psychotherapy**	**0.33**	**0.14**	**2.92**	**0.05**	**0.61**	**0.02**
Somatisation (PHQ−15)	**Time: T0–T1**	**−1.38**	**0.52**	**−2.64**	**−2.4**	**−0.35**	**0.009**
	**Time: T0–T2**	**−2.13**	**0.62**	**−3.42**	**−3.36**	**−0.91**	**0.0008**
	Group	−.29	0.83	−0.35	−1.92	1.33	0.73
	Time (T0–T1) x group	−0.02	0.71	0−.03	−1.4	1.36	0.98
	Time (T0–T2) x group	0.88	0.83	1.06	−0.75	2.5	0.29
	Symptom duration	0.41	0.50	0.81	−0.58	1.39	0.42
	**Depressive disorder**	**2.88**	**0.91**	**3.16**	**1.09**	**4.67**	**0.001**
	Anxiety disorder	0.29	0.86	0.34	−1.4	1.98	0.73
	Previous psychotherapy	1.64	0.90	1.82	−0.13	3.41	0.07
Physical HRQoL (SF−12)	Time: T0–T1	2.10	1.13	1.86	−0.12	4.32	0.07
	**Time: T0–T2**	**6.03**	**1.54**	**3.92**	**3.01**	**9.04**	**<0.001**
	Group	3.31	1.77	1.87	−0.15	6.77	0.06
	Time (T0–T1) x group	−0.16	1.57	−0.10	−3.24	2.92	0.92
	**Time (T0–T2) x group**	**−5.75**	**2.11**	**−2.73**	**−9.88**	**−1.62**	**0.01**
	Symptom duration	−0.93	0.92	−1.02	−2.73	0.87	0.31
	Depressive disorder	−0.65	1.73	−0.38	−4.04	2.74	0.71
	Anxiety disorder	1.88	1.58	1.19	−1.22	4.98	0.24
	Previous psychotherapy	−0.86	1.74	−0.49	−4.28	2.56	0.62
Mental HRQoL (SF−12)	Time: T0–T1	0.66	1.55	0.42	−2.38	3.69	0.67
	Time: T0–T2	2.95	1.80	1.64	−0.59	6.48	0.10
	Group	−0.45	2.14	−0.21	−4.64	3.75	0.83
	Time (T0–T1) x group	3.31	2.15	1.54	−0.91	7.52	0.13
	Time (T0–T2) x group	2.06	2.48	0.83	−2.79	6.91	0.41
	Symptom duration	0.07	1.08	0.07	−2.04	2.18	0.95
	**Depressive disorder**	**−8.32**	**2.03**	**−4.11**	**−12.30**	**−4.35**	**<0.001**
	Anxiety disorder	−1.74	1.85	−0.94	−5.37	1.89	0.35
	**Previous psychotherapy**	**−6.61**	**2.04**	**−3.24**	**−10.60**	**−2.61**	**0.002**
Depression (BDI−II)	Time: T0–T1	−1.75	0.95	−1.84	−3.61	0.11	0.07
	**Time: T0–T2**	**−2.85**	**1.04**	**−2.73**	**−4.90**	**−0.80**	**0.01**
	Group	−1.86	1.44	−1.29	4.69	0.96	0.20
	Time (T0–T1) x group	−0.55	1.27	−0.43	−3.05	1.95	0.67
	Time (T0–T2) x group	0.07	1.39	0.05	−2.67	2.80	0.96
	Symptom duration	0.79	0.83	0.95	−0.84	2.42	0.34
	**Depressive disorder**	**9.08**	**1.54**	**5.91**	**6.07**	**12.09**	**<0.001**
	Anxiety disorder	2.42	1.43	1.69	−0.39	5.23	0.09
	**Previous psychotherapy**	**3.85**	**1.50**	**2.58**	**0.92**	**6.79**	**0.01**
Anxiety (BAI)	Time: T0–T1	−1.66	1.20	−1.39	−4.01	0.69	0.17
	**Time: T0–T2**	**−5.00**	**1.33**	**−3.76**	**−7.60**	**−2.39**	**<0.001**
	Group	0.18	1.91	0.10	−3.56	3.93	0.92
	Time (T0–T1) x group	−0.39	1.62	−0.24	−3.56	2.78	0.81
	Time (T0–T2) x group	2.49	1.77	1.40	−0.99	5.96	0.16
	Symptom duration	1.01	1.12	0.90	−1.18	3.19	0.37
	**Depressive disorder**	**6.24**	**2.06**	**3.04**	**2.21**	**10.28**	**0.003**
	Anxiety disorder	2.25	1.93	1.17	−1.52	6.03	0.24
	**Previous psychotherapy**	**5.10**	**2.01**	**2.54**	**1.16**	**9.03**	**0.01**

Note: All analyses control for age, gender, education, and drop-out; results are not statistically significant for these variables. Time is dummy-coded with Time: T0–T1 representing scores at baseline and after treatment and Time: T0–T2 representing scores at baseline and at 12-months follow-up. Categorical variables are dummy-coded with 0 = self-help group and 1 = treatment group for group, 0 = no and 1 = yes for depressive disorder, anxiety disorder, and previous psychotherapy. For education and symptom duration, higher values mean higher education or longer duration, respectively. Variables printed in bold are significant at an alpha-level of 5%. VHQ = Vertigo Handicap Questionnaire, VSS = Vertigo Symptom Scale, PHQ = Patient Health Questionnaire, SF-12 = Short-Form Health Survey, BDI-II = Beck Depression Inventory-II, BAI = Beck Anxiety Inventory, HRQoL = Health-related quality of life.

## Data Availability

The corresponding author can be contacted to request the data.
